# Transformer-based structuring of free-text radiology report databases

**DOI:** 10.1007/s00330-023-09526-y

**Published:** 2023-03-11

**Authors:** S. Nowak, D. Biesner, Y. C. Layer, M. Theis, H. Schneider, W. Block, B. Wulff, U. I. Attenberger, R. Sifa, A. M. Sprinkart

**Affiliations:** 1 Department of Diagnostic and Interventional Radiology, University Hospital Bonn, Venusberg-Campus 1, 53127 Bonn, Germany; 2 Fraunhofer Institute for Intelligent Analysis and Information Systems IAIS, Sankt Augustin, Germany

**Keywords:** Radiology, Deep learning, Natural language processing, Intensive care units, Thorax

## Abstract

**Objectives:**

To provide insights for on-site development of transformer-based structuring of free-text report databases by investigating different labeling and pre-training strategies.

**Methods:**

A total of 93,368 German chest X-ray reports from 20,912 intensive care unit (ICU) patients were included. Two labeling strategies were investigated to tag six findings of the attending radiologist. First, a system based on human-defined rules was applied for annotation of all reports (termed “silver labels”). Second, 18,000 reports were manually annotated in 197 h (termed “gold labels”) of which 10% were used for testing. An on-site pre-trained model (T_mlm_) using masked-language modeling (MLM) was compared to a public, medically pre-trained model (T_med_). Both models were fine-tuned on silver labels only, gold labels only, and first with silver and then gold labels (hybrid training) for text classification, using varying numbers (*N*: 500, 1000, 2000, 3500, 7000, 14,580) of gold labels. Macro-averaged F1-scores (MAF1) in percent were calculated with 95% confidence intervals (CI).

**Results:**

T_mlm,gold_ (95.5 [94.5–96.3]) showed significantly higher MAF1 than T_med,silver_ (75.0 [73.4–76.5]) and T_mlm,silver_ (75.2 [73.6–76.7]), but not significantly higher MAF1 than T_med,gold_ (94.7 [93.6–95.6]), T_med,hybrid_ (94.9 [93.9–95.8]), and T_mlm,hybrid_ (95.2 [94.3–96.0]). When using 7000 or less gold-labeled reports, T_mlm,gold_ (*N*: 7000, 94.7 [93.5–95.7]) showed significantly higher MAF1 than T_med,gold_ (*N*: 7000, 91.5 [90.0–92.8]). With at least 2000 gold-labeled reports, utilizing silver labels did not lead to significant improvement of T_mlm,hybrid_ (*N*: 2000, 91.8 [90.4–93.2]) over T_mlm,gold_ (*N*: 2000, 91.4 [89.9–92.8]).

**Conclusions:**

Custom pre-training of transformers and fine-tuning on manual annotations promises to be an efficient strategy to unlock report databases for data-driven medicine.

**Key Points:**

• *On-site development of natural language processing methods that retrospectively unlock free-text databases of radiology clinics for data-driven medicine is of great interest.*

• *For clinics seeking to develop methods on-site for retrospective structuring of a report database of a certain department, it remains unclear which of previously proposed strategies for labeling reports and pre-training models is the most appropriate in context of, e.g., available annotator time.*

• *Using a custom pre-trained transformer model, along with a little annotation effort, promises to be an efficient way to retrospectively structure radiological databases, even if not millions of reports are available for pre-training.*

**Supplementary Information:**

The online version contains supplementary material available at 10.1007/s00330-023-09526-y.

## Introduction

Structured reporting, i.e., the use of IT-based systems for importing and arranging medical content in radiological reports, not only has the potential to have a positive impact on patient care by enhancing the quality of radiologists’ practices, it is also beneficial for the development of image-based artificial intelligence systems by helping to compile large retrospective patient collectives with diseases of interest [1, 2]. To this day, most radiological reports are in free-text and not in structured format. Even if a clinic would introduce structured reporting, retrospective assembling of large image collectives is labor-intensive, as the corresponding reports of recent years remain unstructured. Therefore, there is a need for automatic natural language processing (NLP) systems that categorize free-text reports in a set of predefined labels, thereby unlocking the corresponding image database for the development of an artificial intelligence–based diagnostic decision system.

To achieve automated analysis of medical text data, various methods with different levels of complexity have been proposed. For example, simple systems based on human-defined rules have been applied to automatically annotate the occurrence of findings in English chest X-ray reports [3]. These rule-based systems have the advantage that the method itself does not require any manually annotated reports. However, the creator of such a system must have considerable expert knowledge about general information, content, and wording of the reports. Moreover, there may still be findings whose appearance and description are subject to great variability, making the establishment of comprehensive rules a difficult task.

On the other hand, there are machine learning (ML)–based methods that have the disadvantage of requiring a large amount of time-consuming manual annotated reports for training. In recent years, transformer models based on the self-attention mechanism have emerged as the state-of-the-art ML–based NLP method, also for medical text data [4–9]. The required amount of annotated data to train a transformer can be reduced by transfer learning, i.e., utilizing the fundamental text comprehension skills of a model that has already been pre-trained on different large public datasets and/or for another task.

In contrast to radiological image datasets, where the appearance of a finding, e.g., a pneumothorax, is independent of the country, the description of the finding in a radiological report can differ substantially, which is most obvious if the countries do not share a common language. For NLP, this limits the development and application of pre-trained models compared to computer vision applications, as, e.g., a model pre-trained on English reports cannot be directly applied to German texts. Moreover, unlike cross-sectional radiological images, text-based medical data contain sensitive information directly linked to personal data. This makes the public sharing of medical text data in compliance with data protection laws highly problematic in many countries [10]. Even when sharing pre-trained parameters of a transformer model trained on sensible data, it also cannot be ensured that data protection laws are met, as it has been shown that information from the training data can be extracted from large pre-trained transformers [11]. There are efforts to automate the de-identification of German text data using ML methods, from medical and other domains. However, currently, these methods cannot guarantee 100% accuracy [10, 12]. Consequently, efficient development of NLP models for structuring radiological reports on-site is of great interest.

Several approaches have already been presented for the on-site development of transformer models based on radiological text data. Two studies that are based on several hundred thousand English-language reports propose to employ a publicly available transformer pre-trained on medical text and to fine-tune that model in a two-step hybrid label approach. With the hybrid approach, the pre-trained model is first adapted to a high number of rule-based annotated text (termed “silver labels”) and then to only a very limited number of manual annotations (termed “gold labels”) [7, 8]. In contrast, another study proposes a custom pre-training of the transformer by MLM and next-sentence prediction using millions of radiology reports and subsequent fine-tuning to only a few gold-labeled reports [9].

However, for clinics seeking to develop NLP models on-site, it remains unclear which of those pre-training and labeling strategies are most appropriate for structuring their free-text radiological report database. First, it is not clear if a custom pre-training of transformer models is also beneficial with a significantly lower number of reports than in above-mentioned studies. Second, it is not clear whether the effort required to create a rule-based system for silver label generation in hybrid training is worthwhile compared to utilizing more gold labels by investing more annotator time. Therefore, the goal of this work is to provide insight and guidance for efficient retrospective structuring of radiological databases by systematically evaluating the performance of publicly available and custom pre-trained text-based transformers with respect to different labeling strategies and human annotation effort.

## Materials and methods

### Dataset and annotation

With institutional review board approval (AZ 411/21), written informed consent was waived, and approved data processing took place on the basis of the Health Data Protection Act North Rhine-Westphalia (GDSG NW) §6 (2) state law NRW. The retrospective dataset includes 93,368 free-text chest X-ray reports of 20,912 ICU patients (age: 62.7 ± 21.4, 8081 female) from University Hospital Bonn that were extracted consecutively from the radiological information system dating between December 2015 and July 2021.

First, 35 labels including not only findings but also further information, e.g., on indication, were defined for systematic annotation of the reports (see Supplement S1). Information on the interpretation of findings was not assessed from the reports. Under the supervision of a radiology resident (Y.C.L.), two medical research assistants labeled this information using the open-source software doccano [13]. The medical research assistants were trained to assign the correct labels based on the context of the free-text reports and not just on individual words. In case of ambiguity, they were instructed to consult the supervisor. Manually annotated gold labels were curated for 18,000 reports including only reports with unique admission numbers. A subset of six findings that are frequently raised during a patient’s ICU stay were selected from the entire label set. This selection was based on frequency of the finding and their clinical relevance and was made prior to NLP development. The NLP models were developed to predict the occurrence of these labels based on the report text via multi-label classification. These labels and their relative appearances within the gold-labeled reports are pulmonary infiltrates (20.0%), pleural effusion (45.6%), pulmonary congestion (34.0%), pneumothorax (3.8%), regular position of the central venous catheter (CVC) (45.8%), and misplaced position of the CVC (8.4%).

**Fig. 1:**
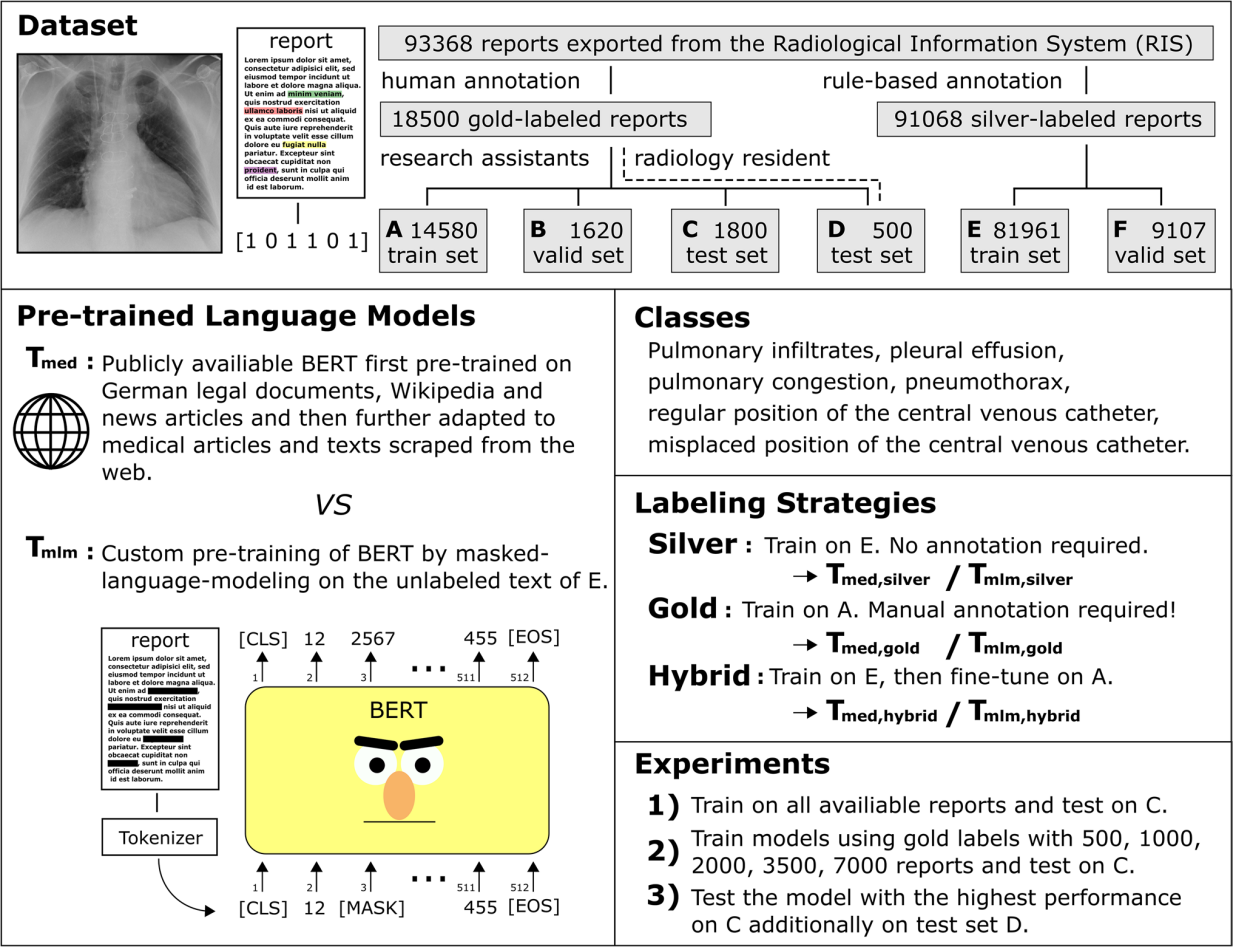
Overview of the presented study. The dataset of the presented study includes a total of 93,368 free-text chest X-ray reports of intensive care unit patients. For a subset of the dataset, human annotations were generated for the occurrence of six findings within the reports to create gold-labeled training, validation, and test datasets. Furthermore, a rule-based system was applied for silver label generation. The use of an on-site pre-trained model using masked-language modeling (T_mlm_) was compared to a public, medically pre-trained model (T_med_) when adapting to silver labels only, gold labels only, and to first with silver and then gold labels (hybrid). To also give insights into which pre-training and labeling strategy is most appropriate in context of available human annotation time, the models were developed using varying numbers of gold-labeled reports

The 18,000 gold-labeled reports were randomly split into training set A (14,580), validation set B (1620), and test set C (1800). Additional 500 reports (test set D) were labeled by the radiology resident and both research assistants independently to determine the agreement between the annotators. This dataset was also used for the final test of the best NLP model with annotations of the radiology resident. Moreover, silver labels were created for a total of 91,068 reports applying a rule-based model which is described below. These are all available reports except the 1800 and 500 texts of the gold-labeled hold-out test sets C and D. Silver-labeled data were split into training set E (81,961) and validation set F (9107). Figure 1 shows an overview of the entire study.

### Rule-based model

A set of rules was defined to automatically annotate the free-text radiological reports. In short, the algorithm searches for specific terms, negations, and descriptions of uncertainty or applies further text-based rules in the “findings” section of the report. Detailed descriptions of the rule-based system can be found in Supplement S2.

### Baseline NLP model

As a baseline NLP approach, we trained a term frequency–inverse document frequency (TFIDF) model on the training text and fitted a one-layer fully connected neural network to the labeled training data [14]. Training details can be found in Supplement S3.

### Transformer-based model

We applied BERT as an established transformer model that has also been used in other work on medical text analysis [5, 7–9]. See Supplement S4 for details on the model architecture.

To investigate the impact of different pre-training strategies, we employed (i) a publicly available BERT language model that was pre-trained on (not annotated) German legal documents, Wikipedia, and news articles and then further adapted to medical articles and texts scraped from the web (T_med_) [15–17] and (ii) created a custom pre-trained BERT language model (T_mlm_) by applying MLM on the texts of train set E. To demonstrate the general effect of pre-training, we also trained a model from scratch for classification on gold-labeled text data without any pre-training (T_rand,gold_).

To examine the difference between different label strategies, three experiments were performed with the two pre-trained models. First, the pre-trained models were fine-tuned on the 14,580 gold labels of training set A only (T_med,gold_, T_mlm,gold_). Second, both pre-trained models were fine-tuned on silver-labeled training set E (T_med,silver_, T_mlm,silver_). Third, the models fine-tuned on silver-labeled training set E (T_med,silver_, T_mlm,silver_) were subsequently fine-tuned on training set A in a hybrid training (T_med,hybrid_, T_mlm,hybrid_). To investigate the effect of the number of available gold labels for fine-tuning, the models were also trained with limited numbers of gold-labeled reports of train set A (500, 1000, 2000, 3500, 7000 reports). All models were tested on test set C, and the best model was additionally tested on test set D.

When fine-tuning the models for text classification, we applied the following concepts. As proposed in previous studies, we fine-tuned all pre-trained models for text classification in two steps: First, frozen pre-trained language model parameters were used to adapt the new classification head and then all parameters were trained, but with layer-specific learning rates with maximum values increasing linearly from 10^−9^ to 10^−6^ from the first to the last layer [18–20]. Since the threshold for binarization of the predictions after sigmoid activation is not intrinsically set in multi-label classification, class-specific thresholds were determined by identifying the thresholds with the highest F1-scores on the training data [21]. Also, oversampling and loss weighting according to the occurrence of the classes within the training data were used to compensate for class imbalance. Classes that occurred in less than 25% of the training reports were duplicated until they accounted for at least 25%.

The pre-trained models fine-tuned for text classification on 14,580 and 7000 gold-labeled reports were trained for 75 epochs. Models that were fine-tuned on less than 7000 reports were trained with the same amount of optimization steps as the models trained on 7000 reports to ensure convergence. The pre-trained models that were fine-tuned on 81,961 silver-labeled reports were trained for 25 epochs.

For the custom pre-training via MLM, first, the BERT model was trained on 81,961 reports with a maximum learning rate of 10^−4^ and 15% of tokens masked for 150 epochs. After that, the model was further trained for 150 epochs with a maximum learning rate of 10^−5^ and 15% of whole words masked within the text. For custom pre-training, no weight decay was used.

For all models, the “bert-base-german-cased” tokenizer of the Huggingface’s transformer library was used and all models were trained using the Adam optimizer with decoupled weight decay regularization of 0.01, a learning rate scheme with warmup until 10% of all training steps and subsequent cosine decay, drop-out of 0.1, mixed precision, random seed 42, and a batch size of 24 on an NVIDIA RTX 3090 or NVIDIA TITAN RTX using PyTorch v1.8.1 and the transformers library v4.13.0 [15, 22]. The performance metrics were calculated using scikit-learn v0.24.2, and 95% CIs were calculated by bootstrapping with 1000 resamples for the text-classification models [23]. Performance differences were considered significant based on non-overlapping CIs.

## Results

Time for manual annotation of 18,000 radiological reports was 197 h (39.4 s per report). The two medical research assistants’ annotations showed high agreement with the radiology resident’s annotations, with mean accuracy of 97.4% and 97.3% and MAF1 in percent of 92.9 and 93.5, respectively.

In the custom pre-training of BERT (T_mlm_), an accuracy of 88.6% was achieved after 3.3 days of training to predict 15% masked tokens and subsequently an accuracy of 78.4% was achieved after again 3.3 days of training to predict 15% masked whole words on test data set C.

Table 1 shows the performance of all examined models trained with all available silver- and/or gold-labeled text classification data. The highest performance was observed for T_mlm,gold_ with a MAF1 in percent of 95.5 (CI: 94.5–96.3), which was significantly higher than that of the rule-based system (75.1 [73.6–76.5]), T_med,silver_ (75.0 [73.4–76.5]), T_mlm,silver_ (75.2 [73.6–76.7]), TFIDF_gold_ (83.2 [81.3–85.1]), and T_rand,gold_ (84.3 [82.5–86.0]). However, the performance was not significantly higher than that of T_med,gold_ (94.7 [93.6–95.6]), T_med,hybrid_ (94.9 [93.9–95.8]), and T_mlm,hybrid_ (95.2 [94.3–96.0]).

**Table 1:** Model performances for different pre-training and labeling strategies using all training data

			Silver		Gold				Hybrid	
Class	SP	RB	T_med_	T_mlm_	TFIDF	T_rand_	T_med_	T_mlm_	T_med_	T_mlm_
Infiltrates	352	69.7	69.3	69.2	79.8	80.3	92.9	92.9	**93.6**	92.0
Congestion	611	94.3	94.1	94.2	88.7	88.2	**98.1**	**98.1**	**98.1**	97.9
Effusion	818	95.0	94.6	94.7	88.7	91.0	**98.8**	**98.8**	**98.8**	**98.8**
Pneumothorax	65	87.8	87.1	87.0	75.2	79.7	96.1	96.0	**98.5**	98.4
Regular CVC	825	67.0	67.8	67.9	89.8	90.7	93.4	**95.4**	94.9	95.0
Misplaced CVC	151	36.9	37.3	38.1	77.2	75.7	88.8	**91.7**	85.6	89.3
Macro average	2822	75.1	75.0	75.2	83.2	84.3	94.7	**95.5**	94.9	95.2
Micro average	2822	77.3	77.3	77.5	87.1	87.7	95.7	**96.5**	96.1	96.1

F1-scores (%) observed for the hold-out test set of 1800 gold-labeled reports for the rule-based (RB) system, the TFIDF approach and the transformer models trained with all 14,580 gold-labeled training data

The support (SP), i.e., the number of positive samples, is given for each class

For each class, the highest F1-scores are indicated in bold

**Fig. 2:**
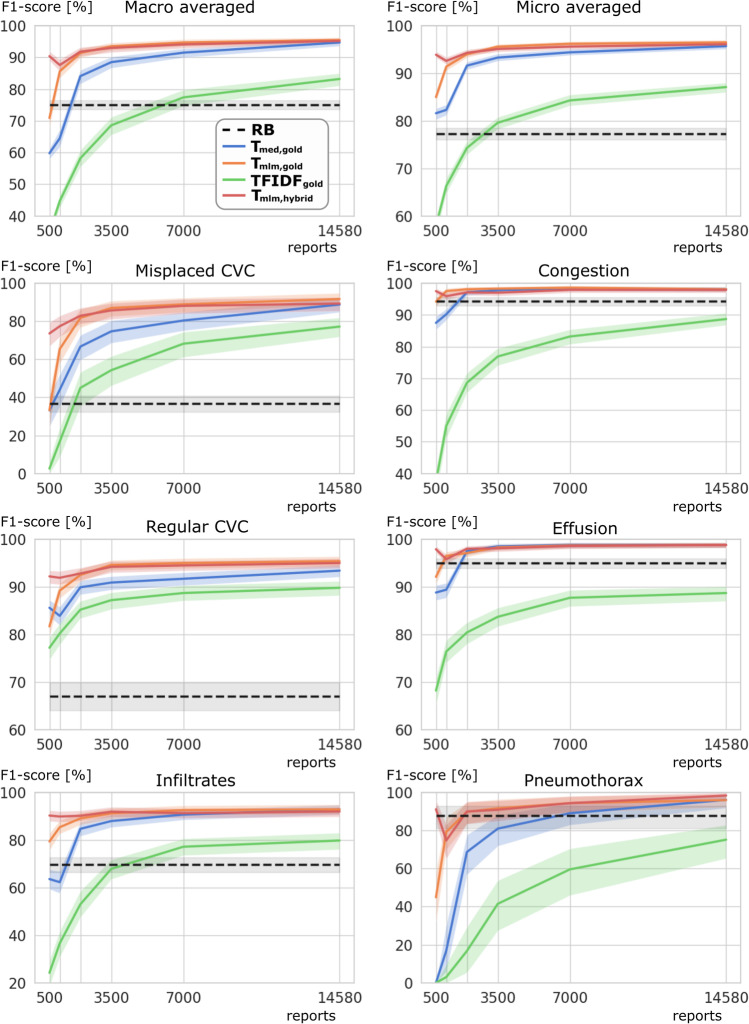
Model performances for different numbers of gold-labeled reports. F1-scores in % (*y*-axis) are displayed for the rule-based (RB) system in black, as well as for T_med,gold_ (blue), T_mlm,gold_ (orange), TFIDF_gold_ (green), and T_mlm,hybrid_ (red) using various numbers of gold-labeled reports for training (*x*-axis)

Table 2 and Fig. 2 show the performance of the examined models for all classes when using lower numbers of gold-labeled data. The classification of the description of a misplaced CVC was found to be the most challenging class with the lowest F1-scores for each method. Considering models trained exclusively with gold-labeled reports, T_mlm,gold_ showed significantly higher MAF1 as well as misplaced CVC F1-scores than TFIDF_gold_ and T_med,gold_ when only 1000 to 7000 gold-labeled reports were provided for training. The hybrid models adapted on only 500 gold labels (T_mlm,hybrid_ 90.4 [89.0–91.9], T_med,hybrid_ 86.9 [85.1–88.5]) achieved already significantly higher MAF1 than the rule-based system (75.1 [73.6–76.5]). Considering both models trained with a hybrid label scheme, no significant differences in MAF1 and F1-scores for misplaced CVC were observed for T_med,hybrid_ and T_mlm,hybrid_ trained with 1000 or more gold labels. When using 2000 or more gold-labeled reports (22 h of annotation), the previous use of silver labels in hybrid training of the BERT models (T_mlm,hybrid_ 91.8 [90.4–93.2], T_med,hybrid_ 89.1 [87.6–90.6]) did not provide a significant improvement of MAF1 over the BERT models (T_mlm,gold_ 91.4 [89.9–92.8], T_med,gold_ 84.1 [81.7, 86.0]) trained directly on the gold-labeled reports. Due to less than 4% positive pneumothorax cases leading to wide CIs, F1-scores for pneumothorax of the rule-based system (87.8 [81.1–93.0]) are only significantly lower compared with T_mlm,hybrid_ (98.4 [95.9–100.0]) and T_med,hybrid_ (98.5 [96.0–100.0]) trained with all gold labels.

**Table 2:** Model performances for different pre-training and labeling strategies using different numbers of gold-labeled training data

*N*		Gold			Hybrid		Gold			Hybrid	
		TFIDF	T_med_	T_mlm_	T_med_	T_mlm_	TFIDF	T_med_	T_mlm_	T_med_	T_mlm_
		Macro averaged					Micro averaged				
500	(3.4%)	34.9	59.8	70.9*	86.9^†^	90.4*^†^	57.9	81.6	85.0*	92.7^†^	**93.9**^†^
1000	(6.9%)	44.7	64.5	85.6*	**91.5**^†^	87.6	66.3	82.3	91.4*	**94.4**^†^	92.6
2000	(13.7%)	58.2	84.1	91.4*	89.1	**91.8**	74.3	91.6	94.0*	93.0	94.3*
3500	(24.0%)	68.6	88.5	93.5*	91.6	93.0	79.6	93.3	95.6*	94.1	95.1
7000	(48.0%)	77.4	91.5	94.7*	92.1	94.1	84.3	94.4	96.2*	94.4	95.6*
14,580	(100%)	83.2	94.7	**95.5**	94.9	95.2	87.1	95.7	**96.5**	96.1	96.1
		Misplaced CVC					Congestion				
500	(3.4%)	2.6	33.2	33.3	53.4^†^	73.6*^†^	37.4	87.5	94.2*	**97.6**^†^	97.5^†^
1000	(6.9%)	16.8	44.0	65.4*	77.1	**77.4**^†^	55.0	90.2	97.5*	**97.9**	95.9
2000	(13.7%)	45.0	66.7	81.8*	72.2	82.9*	68.6	97.1	**98.1**	97.2	97.1
3500	(24.0%)	54.3	74.7	87.0*	78.0	85.7	76.9	97.8	**98.3**	97.1	97.0
7000	(48.0%)	68.2	80.4	88.9*	80.9	88.1	83.2	98.4	**98.6**	97.9	97.9
14,580	(100%)	77.2	88.8	**91.7**	85.6	89.3	88.7	**98.1**	**98.1**	**98.1**	97.9
		Regular CVC					Effusion				
500	(3.4%)	77.2	85.6	81.7	90.9^†^	**92.2**^†^	68.2	88.8	92.1*	**98.3**^†^	97.9^†^
1000	(6.9%)	80.2	83.9	89.2*	**92.7**^†^	91.9	76.4	89.4	96.5*	**98.5**^†^	95.8
2000	(13.7%)	85.2	89.9	92.5	92.4	**92.8**	80.4	97.5	97.1	97.9	**98.0**
3500	(24.0%)	87.2	90.9	94.6*	92.3	94.2	83.7	**98.5**	98.3	98.4	98.1
7000	(48.0%)	88.7	91.7	95.0*	93.1	94.5	87.7	**98.8**	98.7	98.2	98.6
14,580	(100%)	89.8	93.4	**95.4**	94.9	95.0	88.7	**98.8**	**98.8**	**98.8**	**98.8**
		Infiltrates					Pneumothorax				
500	(3.4%)	24.2	63.6	79.4*	88.0^†^	**90.3**^†^	0.0	0.0	44.9*	**93.0**^†^	91.2^†^
1000	(6.9%)	36.6	62.3	85.4*	**90.6**^†^	89.9	3.0	16.9	79.3*	**92.2**^†^	74.8
2000	(13.7%)	53.0	84.7	89.1	86.5	**90.2**	16.7	68.7	89.8*	88.7	**90.0**
3500	(24.0%)	67.9	88.0	91.2	90.5	**91.9**	41.5	81.0	91.7	**93.7**	90.9
7000	(48.0%)	77.2	90.7	**92.6**	89.4	91.3	59.6	89.1	**94.4**	93.1	**94.4**
14,580	(100%)	79.8	92.9	92.9	**93.6**	92.0	75.2	96.1	96.0	**98.5**	98.4

F1-scores (%) observed on all classes for the hold-out test set of 1800 gold-labeled reports for the experiments on training with the different numbers (*N*) of the 14,580 gold-labeled training reports. The highest F1-scores of a class at a given *N* are highlighted by bold font. Approximately 5.5 h of work was performed to annotate 500 reports

^*^Significantly higher F1-scores comparing to all models trained with the same label strategy (gold or hybrid), independent of the model (T_med_, T_mlm_, TFIDF)

^†^Significantly higher F1-scores of a hybrid or gold-trained model respectively compared to all models trained with the other label strategy

Table 3 shows further performance metrics for the best model, T_mlm,gold_, which was pre-trained using MLM and fine-tuned to 14,580 gold labels that showed the highest MAF1 (95.5, CI: 94.5–96.3) and macro-averaged area under the receiver operating characteristic curve (MAAUC: 97.1, CI: 96.3–97.8) for test set C with 1800 cases, as well as for test set D with 500 cases (MAF1: 93.5, CI: 91.0–95.3; MAAUC: 95.7, CI: 93.8–97.1). Due to space limitations, CIs for each single value in Tables 1–3 can be found in Supplement S5.

**Table 3:** Detailed model performance of T_mlm,gold_ for both test sets

Class	1800 test set						500 test set					
	SP	AC	PR	RC	F1	AUC	SP	AC	PR	RC	F1	AUC
Infiltrates	352	97.2	91.2	94.6	92.9	96.2	105	94.6	86.1	88.6	87.3	92.4
Congestion	611	98.7	97.6	98.7	98.1	98.7	176	97.8	97.1	96.6	96.9	97.5
Effusion	818	98.9	98.5	99.0	98.8	98.9	241	99.4	100	98.8	99.4	99.4
Pneumothorax	65	99.7	100	92.3	96.0	96.2	20	99.4	100	85.0	91.9	92.5
Regular CVC	825	95.8	95.8	95.0	95.4	95.8	211	95.8	93.6	96.7	95.1	95.9
Misplaced CVC	151	98.6	88.8	94.7	91.7	96.8	50	98.0	87.0	94.0	90.4	96.2
Macro average	2822	98.1	95.3	95.7	95.5	97.1	803	97.5	94.0	93.3	93.5	95.7
Micro average	2822	97.7	96.1	96.8	96.5	97.4	803	97.4	95.1	95.8	95.4	96.8

F1-scores and AUC in % for each class on both test sets for T_mlm,gold_ trained with all available data

The support (SP), i.e., the number of positive samples is given for each class and both test sets

*AC* accuracy, *RC* recall, *PR* precision

## Discussion

The current study investigates efficient on-site development of NLP methods in the context of different pre-training and labeling strategies for structuring and thus unlocking radiological databases for data-driven medicine using German ICU chest X-ray reports. The work provides clinics seeking to develop NLP models on-site with insights and guidance on which strategy is preferable for their specific project in the context of available annotator and developer time and the complexity of the information to be extracted. Methods for training transform-based NLP models will be provided upon reasonable request (https://qilab.de).

The results show that when training with a large set of silver labels without the use of gold labels, the pre-trained BERT models achieved comparable performance to the rule-based system and are limited by the quality of the silver labels. By using a publicly available, medically pre-trained BERT with a hybrid label approach that was first adapted on all silver labels and then fine-tuned on a small set of gold-labeled reports, significantly higher performance can be achieved compared to the rule-based system. Both findings are in line with previous studies that used silver-labeled chest X-ray reports in the English language by CheXpert and further trained on 1000 manually curated reports or sentences [3, 7, 8]. However, when utilizing 2000 or more gold-labeled reports that were generated in only 22 h of annotation, the hybrid label approach did not provide a significant improvement over training the publicly available BERT model directly with the gold labels. The results of this work also show that the custom pre-training of BERT with only 81,961 reports can not only achieve high MLM accuracy compared to previous studies with millions of reports [9], but also demonstrate that this model achieves significantly higher performance than the publicly available pre-trained BERT model when further trained for report classification with 7000 or less gold labels.

The performance of the rule-based system that generates the silver labels varies strongly between the extracted classes. This can be explained by the fact that some information from the radiological reports have more variable attributes and are more difficult to identify by rules and standard formulations. In contrast to previous studies, we extracted information about a regular or misplaced position of the CVC from the findings. Especially, the formulations for a misplaced CVC appeared to be difficult to recognize with simple rules, in contrast to, e.g., pleural effusion and pulmonary congestion. Through further effort by clinicians and technicians, certainly more special cases could be covered by advanced rules, in order to further develop the simple rule-based labeler. However, this would require extensive reading and studying of the reports, during which gold labels could already be generated. With regard to the results of this study, it is therefore questionable whether for information with variable attributes and descriptions, the effort of developing advanced rules for a rule-based labeler is worthwhile compared to generating more gold labels with subsequent training of custom pre-trained transformers.

A limitation of the study is that annotation of the contents of the radiology reports was performed by medical research assistants under the supervision of a radiology resident. Because the annotators did not have to interpret imaging, but were simply required to identify and mark the statements of the attending radiologist within the report, we judged that annotation was not required to be conducted by board-certified radiologists. The high agreement of the different annotators confirmed this judgement, which minimized the cost of annotation and allowed for capturing a larger set of reports.

## Conclusion

In conclusion, we find that an on-site custom pre-training of text-based transformers with subsequent adaptation to manually curated gold labels promises to be an efficient strategy to unlock radiological report databases for data-driven medicine.

## Supplementary Information

Below is the link to the electronic supplementary material.

Supplementary file1 (DOCX 52.2 KB)

## Abbreviations

CI: Confidence interval
CVC: Central venous catheter
ICU: Intensive care unit
MAAUC: Macro-averaged area under the receiver operating characteristic curve
MAF1: Macro-averaged F1-score
ML: Machine learning
MLM: Masked-language modeling
NLP: Natural language processing
TFIDF: Term frequency–inverse document frequency

## Declarations

### Guarantor

The scientific guarantor of this publication is PD Dr.-Ing. Alois Martin Sprinkart.

### Statistics and biometry

No complex statistical methods were necessary for this paper.

### Methodology

• retrospective

• experimental study

• performed at one institution
