# Supplementary material for: Transformer-based structuring of free-text radiology report databases
Source: Eur Radiol. 2023 Mar 11;33(6):4228–36. doi: 10.1007/s00330-023-09526-y (PMC10181962; doi:10.1007/s00330-023-09526-y)
Supplement: Supplementary file 1 — Supplementary file1 (DOCX 52.2 KB) [file 330_2023_9526_MOESM1_ESM.docx]

**Supplemental Materials**

**S1 Complete Labeling System**

The complete label system for chest X-ray reports of the intensive care unit of our hospital is structured as follows:

- *Identifying information*: Names, dates
- *Indications*:
  - pulmonary infiltrates
  - pleural effusion
  - pulmonary congestion
  - pneumothorax
  - material position
  - other
- *Findings*:
  - pulmonary infiltrates
  - pleural effusion
  - pulmonary congestion
  - pneumothorax
  - atelectasis
  - soft tissue emphysema
  - mediastinal emphysema
  - mediastinal shift
  - fracture
  - other extrathoracic secondary findings
- *Material positions* (regular/misplaced):
  - central venous catheter (CVC)
  - pleural drainage
  - endotracheal tube
  - tracheal cannula
  - gastric tube
  - cardiac pacemaker
  - other
- *Image quality*:
  - image truncated
  - image skewed

For gold-labeling, the reports were split into work packages of 500 reports and imported as sequence labeling projects in the open-source software doccano [1]. Each project was assigned to a single annotator with exception of a work package of 500 representing the test set D for rater agreement analysis which was labeled by both medical research assistances and the radiology resident.

Using doccano, the annotators marked the relevant information in the text and selected the corresponding label class. In order to make the workflow more efficient and to anonymize the reports automatically as much as possible, the patient name and all data in the format ‘dd.mm.yyyy’ were automatically searched for, annotated and replaced with Ps or Ds in each finding. Furthermore, an early version of the rule-based labeler was used to set marks for some classes, which the annotators clicked off or re-set as needed.

**S2 Procedure of the rule-based model**

First, the rule-based labeling algorithm searched for the occurrence of following specific terms:

- *Pulmonary infiltrates*: Infiltrat(e) (English: infiltrate(s))
- *Pleural effusion*: Pleuraerguss, Pleuraergüsse, Erguss, Ergüsse (English: pleural effusion(s), effusion(s))
- *Pulmonary congestion*: Stauung, stauungsbedingt (English: congestion, congestion-related)
- *Pneumothorax*: pneumothorax, pneumothoraces
- *Regular/non-regular position of CVC*: zentraler Venenkatheter, ZVK (English: central venous catheter, CVC)

The relevant part of the sentence with regard to the term was identified by splitting the reports at punctuation marks (.,:,?,!) and occurrences of terms of another class. If the sentence is not in the ‘Findings’ section of the report or if there is a question mark at the end of the sentence, the word was not labeled. For all classes, except the classes regarding the location of the CVC, the relevant part of the sentence is searched for a negation (kein, keine, ohne, nicht; English: no, none, without, not) or a description of uncertainty (nicht ausgeschlossen / ausschließen / auszuschließen; English: not excluded).

If no negation or description of uncertainty is found, the term was labeled with the corresponding class.

For the position of the CVC, standard descriptions for a regular position (regelrechte Lage, regelrechter Projektion, regelhafter Lage, regelhafter Projektion; English: regular position, regular projection) or an incorrect position (Fehllage; English: incorrect position) are searched for and labeled accordingly. If no standard description for the position was found, then a search is made for a mention of the Vena Cava Superior (VCS) in the relevant part of the sentence. If the VCS was mentioned, then a regular position is assumed, otherwise a non-regular position.

**S3 TF-IDF training**

A TF-IDF model analyses a set of text data and extracts the most significant n-grams defining that allow for differentiation between texts. The output of a TF-IDF model is a vector for each document (i.e. radiological report), each vector element describing the term-frequency of a vocabulary item in the document divided by the document frequency of the vocabulary item in the training corpus.

We train TF-IDF model with a set vocabulary size of 25000 and a n-gram range of 1 to 3. We preprocess the training text by removing numbers, removing German stop words, ignoring casing, and stemming each token using the German SnowballStemmer of the nltk library [2,3]. For classification, we train a one-layer fully connected neural network, i.e. a linear model for each label, until convergence on the labeled training dataset.

**S4 BERT Transformer model**

The model consists of two parts: a language model that reads and reconstructs a given text sequence and a classification layer (a single linear layer with sigmoid activations) that predicts labels from the latent representation of the sequence provided by the language model. The language model can be pre-trained on non-annotated raw text data, the classification layer requires labeled data for training. To tokenize text, BERT employs sub word tokenization [4].

Fine-tuning a pre-trained model then is continuing the model training on the annotated data, which adapts both the language model and the classification layer to the new data domain. We employ the BERT configuration from Huggingface's transformers library [5-7] with a vocab size of 30000, hidden size 768, 12 hidden layer with 12 attention heads each for a total number of 110M parameters.

**S5 Tables with confidence intervals**

|  |  |  |  | silver | |  |  |  | gold | |  |  |  |  | hybrid |
| --- | --- | --- | --- | --- | --- | --- | --- | --- | --- | --- | --- | --- | --- | --- | --- |
| Class | SP | RB | T_med_ | | T_mlm_ | | TFIDF | | T_rand_ | T_med_ | T_mlm_ | | T_med_ | | T_mlm_ |
| Infiltrates | 352 | 69.7  [66.5-72.7] | 69.3  [66.0-72.5] | | 69.2  [65.8-72.3] | | 79.8*  [76.6-82.9] | | 80.3*  [77.1-83.3] | 92.9*  [90.7-94.8] | 92.9*  [90.8-94.9] | | **93.6***  [91.5-95.3] | | 92.0*  [89.8-93.9] |
| Congestion | 611 | 94.3  [93.0-95.5] | 94.1  [92.7-95.4] | | 94.2  [92.8-95.5] | | 88.7  [86.8-90.5] | | 88.2  [86.2-90.0] | **98.1***  [97.3-98.8] | **98.1***  [97.4-98.8] | | **98.1***  [97.3-98.8] | | 97.9*  [97.0-98.6] |
| Effusion | 818 | 95.0  [93.9-96.0] | 94.6  [93.5-95.7] | | 94.7  [93.7-95.7] | | 88.7  [87.1-90.3] | | 91.0  [89.5-92.3] | **98.8***  [98.2-99.3] | **98.8***  [98.2-99.3] | | **98.8***  [98.2-99.3] | | **98.8***  [98.2-99.3] |
| Pneumothorax | 65 | 87.8  [81.1-93.0] | 87.1  [81.2-92.5] | | 87.0  [80.8-92.3] | | 75.2  [66.0-83.3] | | 79.7  [71.2-86.9] | 96.1  [91.9-99.1] | 96.0  [92.3-99.1] | | **98.5***  [96.0-100.0] | | 98.4*  [95.9-100.0] |
| Regular CVC | 825 | 67.0  [64.0-69.8] | 67.8  [64.6-70.5] | | 67.9  [64.5-70.9] | | 89.8*  [88.2-91.3] | | 90.7*  [89.2-92.2] | 93.4*  [92.1-94.6] | **95.4***  [94.3-96.4] | | 94.9*  [93.8-95.9] | | 95.0*  [93.9-96.0] |
| Misplaced CVC | 151 | 36.9  [32.3-40.8] | 37.3  [32.7-41.3] | | 38.1  [33.5-42.5] | | 77.2*  [72.0-82.4] | | 75.7*  [70.4-80.8] | 88.8*  [84.8-92.6] | **91.7***  [88.5-94.8] | | 85.6*  [81.0-89.8] | | 89.3*  [85.4-92.7] |
| Macro average | 2822 | 75.1  [73.6-76.5] | 75.0  [73.4-76.5] | | 75.2  [73.6-76.7] | | 83.2*  [81.3-85.1] | | 84.3*  [82.5-86.0] | 94.7*  [93.6-95.6] | **95.5***  [94.5-96.3] | | 94.9*  [93.9-95.8] | | 95.2*  [94.3-96.0] |
| Micro average | 2822 | 77.3  [76.0-78.5] | 77.3  [76.0-78.6] | | 77.5  [76.2-78.7] | | 87.1*  [86.0-88.1] | | 87.7*  [86.8-88.7] | 95.7*  [95.1-96.3] | **96.5***  [95.9-97.0] | | 96.1*  [95.5-96.7] | | 96.1*  [95.5-96.6] |

**Table 1.** Top: F1-scores (%) observed for the hold-out test set of 1800 gold-labeled reports for the rule-based (RB) system, the TFIDF approach and the transformer models trained with all 14580 gold-labeled training data. The support (SP), i.e. the number of positive samples is given for each class. Significantly higher F1 values compared to the rule-based system for a given class are indicated by *.

|  |  |  |  | gold |  |  |  | hybrid | |  |  |  | gold |  |  |  |  | hybrid |
| --- | --- | --- | --- | --- | --- | --- | --- | --- | --- | --- | --- | --- | --- | --- | --- | --- | --- | --- |
| N |  | TFIDF | | T_med_ | T_mlm_ | | T_med_ | | T_mlm_ | | TFIDF | | T_med_ | T_mlm_ | | T_med_ | | T_mlm_ |
|  |  | Macro averaged | | | | | | | | | Micro averaged | | | | | | | |
| 500 | (3.4%) | 34.9  [33.5-36.3] | | 59.8  [58.0-61.3] | 70.9*  [68.0-73.6] | | 86.9†  [85.1-88.5] | | **90.4***†  [89.0-91.9] | | 57.9  [56.0-59.5] | | 81.6  [80.3-82.6] | 85.0*  [84.0-86.0] | | 92.7†  [92.0-93.5] | | **93.9**†  [93.3-94.6] |
| 1000 | (6.9%) | 44.7  [42.6-46.6] | | 64.5  [61.8-67.2] | 85.6*  [83.5-87.4] | | **91.5**†  [90.2-92.8] | | 87.6  [85.7-89.4] | | 66.3  [64.8-68.0] | | 82.3  [81.2-83.4] | 91.4*  [90.6-92.2] | | **94.4**†  [93.8-95.1] | | 92.6  [91.9-93.4] |
| 2000 | (13.7%) | 58.2  [55.5-60.8] | | 84.1  [81.7-86.0] | 91.4*  [89.9-92.8] | | 89.1  [87.6-90.6] | | **91.8**  **[90.4-93.2]** | | 74.3  [72.9-75.7] | | 91.6  [90.8-92.4] | 94.0*  [93.3-94.6] | | 93.0  [92.3-93.7] | | **94.3***  [93.7-95.0] |
| 3500 | (24.0%) | 68.6  [65.6-71.2] | | 88.5  [86.7-90.2] | **93.5***  [92.3-94.6] | | 91.6  [90.3-92.8] | | 93.0  [91.6-94.1] | | 79.6  [78.3-80.8] | | 93.3  [92.6-94.1] | **95.6***  [95.0-96.1] | | 94.1  [93.4-94.8] | | 95.1  [94.5-95.7] |
| 7000 | (48.0%) | 77.4  [74.8-79.8] | | 91.5  [90.0-92.8] | **94.7***  [93.5-95.7] | | 92.1  [90.8-93.2] | | 94.1  [92.9-95.2] | | 84.3  [83.2-85.5] | | 94.4  [93.8-95.1] | **96.2***  [95.7-96.7] | | 94.4  [93.8-95.0] | | 95.6*  [95.0-96.2] |
| 14580 | (100%) | 83.2  [81.1-85.0] | | 94.7  [93.6-95.6] | **95.5**  [94.5-96.3] | | 94.9  [93.9-95.8] | | 95.2  [94.3-96.0] | | 87.1  [86.0-88.0] | | 95.7  [95.1-96.3] | **96.5**  **[95.9-97.0]** | | 96.1  [95.5-96.7] | | 96.1  [95.5-96.6] |
|  |  | Misplaced CVC | | | | | | | | | Congestion | | | | | | | |
| 500 | (3.4%) | 2.6  [0.0-6.9] | | 33.2  [24.6-41.5] | 33.3  [23.4-41.9] | | 53.4†  [44.7-61.2] | | **73.6***†  [66.9-79.4] | | 37.4  [33.1-41.0] | | 87.5  [85.4-89.5] | 94.2*  [92.7-95.6] | | **97.6**†  [96.8-98.4] | | 97.5†  [96.5-98.3] |
| 1000 | (6.9%) | 16.8  [8.6-24.0] | | 44.0  [35.2-52.1] | 65.4*  [57.9-71.7] | | 77.1  [71.4-82.4] | | **77.4**†  [71.8-82.9] | | 55.0  [51.2-59.0] | | 90.2  [88.4-91.9] | 97.5*  [96.6-98.4] | | **97.9**  [97.1-98.7] | | 95.9  [94.8-97.0] |
| 2000 | (13.7%) | 45.0  [35.3-53.3] | | 66.7  [59.7-72.7] | 81.8*  [76.8-86.4] | | 72.2  [66.7-77.7] | | **82.9***  [78.5-87.0] | | 68.6  [65.5-71.7] | | 97.1  [96.1-98.0] | **98.1**  [97.3-98.8] | | 97.2  [96.2-98.1] | | 97.1  [96.3-98.1] |
| 3500 | (24.0%) | 54.3  [46.2-61.6] | | 74.7  [68.6-80.4] | **87.0***  [82.6-90.7] | | 78.0  [72.9-82.4] | | 85.7  [81.1-89.6] | | 76.9  [74.1-79.5] | | 97.8  [96.9-98.6] | **98.3**  [97.5-98.9] | | 97.1  [96.0-98.0] | | 97.0  [96.0-98.0] |
| 7000 | (48.0%) | 68.2  [61.2-74.4] | | 80.4  [75.1-84.8] | **88.9***  [84.8-92.3] | | 80.9  [76.1-85.2] | | 88.1  [84.0-91.7] | | 83.2  [80.8-85.4] | | 98.4  [97.6-99.0] | **98.6**  [97.9-99.2] | | 97.9  [97.0-98.6] | | 97.9  [97.0-98.7] |
| 14580 | (100%) | 77.2  [71.9-81.9] | | 88.8  [84.8-92.6] | **91.7**  [88.5-94.8] | | 85.6  [81.0-89.8] | | 89.3  [85.4-92.7] | | 88.7  [86.8-90.6] | | **98.1**  [97.3-98.8] | **98.1**  [97.4-98.8] | | **98.1**  [97.3-98.8] | | 97.9  [97.0-98.6] |
|  |  |  | | | | | | | | |  | | | | | | | |

|  |  | Regular CVC | | | | | Effusion | | | | |
| --- | --- | --- | --- | --- | --- | --- | --- | --- | --- | --- | --- |
| 500 | (3.4%) | 77.2  [74.7-79.4] | 85.6  [83.8-87.2] | 81.7  [79.6-83.7] | 90.9†  [89.4-92.2] | **92.2**†  [90.7-93.4] | 68.2  [65.3-70.9] | 88.8  [87.2-90.3] | 92.1*  [90.6-93.4] | **98.3**†  [97.6-98.9] | 97.9†  [97.2-98.6] |
| 1000 | (6.9%) | 80.2  [77.9-82.3] | 83.9  [81.9-85.8] | 89.2*  [87.6-90.8] | **92.7**†  [91.3-93.9] | 91.9  [90.5-93.3] | 76.4  [74.1-78.8] | 89.4 [  87.7-90.8] | 96.5*  [95.7-97.4] | **98.5**†  [97.9-99.0] | 95.8  [94.8-96.8] |
| 2000 | (13.7%) | 85.2  [83.4-87.0] | 89.9  [88.4-91.4] | 92.5  [91.1-93.8] | 92.4  [91.0-93.7] | **92.8**  [91.4-94.0] | 80.4  [78.0-82.5] | 97.5  [96.7-98.2] | 97.1  [96.3-97.9] | 97.9  [97.2-98.5] | **98.0**  [97.3-98.6] |
| 3500 | (24.0%) | 87.2  [85.3-88.8] | 90.9  [89.4-92.2] | **94.6***  [93.5-95.6] | 92.3  [90.7-93.6] | 94.2  [93.0-95.4] | 83.7  [81.7-85.6] | **98.5**  [97.9-99.1] | 98.3  [97.7-98.9] | 98.4  [97.7-98.9] | 98.1  [97.4-98.8] |
| 7000 | (48.0%) | 88.7  [87.1-90.3] | 91.7  [90.3-93.0] | **95.0***  [93.9-96.0] | 93.1  [91.8-94.3] | 94.5  [93.4-95.5] | 87.7  [85.9-89.3] | **98.8**  [98.2-99.3] | 98.7  [98.1-99.3] | 98.2  [97.5-98.8] | 98.6  [98.0-99.2] |
| 14580 | (100%) | 89.8  [88.2-91.2] | 93.4  [92.1-94.6] | **95.4**  [94.3-96.4] | 94.9  [93.8-95.9] | 95.0  [93.9-96.0] | 88.7  [87.0-90.1] | **98.8**  [98.2-99.3] | **98.8**  [98.2-99.3] | **98.8**  [98.2-99.3] | **98.8**  [98.2-99.3] |
|  |  | Infiltrates | | | | | Pneumothorax | | | | |
| 500 | (3.4%) | 24.2  [18.6-29.5] | 63.6  [59.4-67.5] | 79.4*  [75.7-82.8] | 88.0†  [85.3-90.5] | **90.3**†  [87.9-92.5] | 0.0  [0.0-0.0] | 0.0  [0.0-0.0] | 44.9*  [31.9-58.0] | **93.0**†  [88.2-97.2] | 91.2†  [85.5-95.9] |
| 1000 | (6.9%) | 36.6  [30.9-41.7] | 62.3  [57.7-66.9] | 85.4*  [82.7-88.2] | **90.6**†  [88.5-92.7] | 89.9  [87.6-92.1] | 3.0  [0.0-9.7] | 16.9  [6.0-30.4] | 79.3*  [70.1-87.0] | **92.2**†  [87.0-96.4] | 74.8  [65.1-83.2] |
| 2000 | (13.7%) | 53.0  [47.6-58.6] | 84.7  [81.7-87.6] | 89.1  [86.4-91.5] | 86.5  [83.7-89.0] | **90.2**  [87.9-92.4] | 16.7  [5.4-29.3] | 68.7  [56.9-77.8] | 89.8*  [83.5-94.9] | 88.7  [82.6-94.3] | **90.0**  [83.5-95.2] |
| 3500 | (24.0%) | 67.9  [63.6-71.8] | 88.0  [85.4-90.6] | 91.2  [88.8-93.3] | 90.5  [88.2-92.7] | **91.9**  [89.7-93.9] | 41.5  [27.5-53.8] | 81.0  [71.9-88.1] | 91.7  [86.3-96.4] | **93.7**  [88.7-97.7] | 90.9  [84.9-95.8] |
| 7000 | (48.0%) | 77.2  [73.6-80.6] | 90.7  [88.5-92.7] | **92.6**  [90.6-94.5] | 89.4  [87.0-91.5] | 91.3  [89.2-93.3] | 59.6  [46.0-70.5] | 89.1  [83.0-94.4] | **94.4**  [89.6-98.1] | 93.1  [88.3-97.2] | **94.4**  [89.6-98.0] |
| 14580 | (100%) | 79.8  [76.1-83.0] | 92.9  [90.7-94.8] | 92.9  [90.8-94.9] | **93.6**  [91.5-95.3] | 92.0  [89.8-93.9] | 75.2  [65.4-82.9] | 96.1  [91.9-99.1] | 96.0  [92.3-99.1] | **98.5**  [96.0-100.0] | 98.4  [95.9-100.0] |

**Table 2.** F1-scores (%) observed on all classes for the hold-out test set of 1800 gold-labeled reports for the experiments on training with the different numbers (N) of the 14580 gold-labeled training reports. The highest F1-scores of a class at a given n are highlighted by bold font. Significantly higher F1-scores comparing all models trained with the same label strategy (gold or hybrid), independent of the model (T_med_, T_mlm_, TFIDF) are indicated by *. Significantly higher F1-scores of a hybrid or gold trained model respectively compared to all models trained with the other label strategy are indicated by †. Approximately 5.5 hours of work was performed to annotate 500 reports.

| test set C (N=1800) | | | | | | |
| --- | --- | --- | --- | --- | --- | --- |
| Class | SP | Accuracy | Precision | Recall | F1-score | AUC |
| Infiltrates | 352 | 97.2 [96.4-98.0] | 91.2 [88.5-94.3] | 94.6 [91.9-97.0] | 92.9 [90.9-94.9] | 96.2 [94.8-97.4] |
| Congestion | 611 | 98.7 [98.2-99.2] | 97.6 [96.3-98.7] | 98.7 [97.8-99.5] | 98.1 [97.3-98.8] | 98.7 [98.2-99.2] |
| Effusion | 818 | 98.9 [98.4-99.3] | 98.5 [97.7-99.3] | 99.0 [98.3-99.6] | 98.8 [98.2-99.3] | 98.9 [98.4-99.3] |
| Pneumothorax | 65 | 99.7 [99.4-99.9] | 100.0 [100-100] | 92.3 [84.6-98.4] | 96.0 [91.7-99.2] | 96.2 [92.3-99.2] |
| Regular CVC | 825 | 95.8 [94.9-96.8] | 95.8 [94.4-97.2] | 95.0 [93.6-96.5] | 95.4 [94.4-96.5] | 95.8 [94.8-96.8] |
| Misplaced CVC | 151 | 98.6 [98.0-99.1] | 88.8 [83.9-93.9] | 94.7 [91.0-98.0] | 91.7 [88.3-94.8] | 96.8 [95.0-98.5] |
| Macro average | 2822 | 98.1 [97.9-98.4] | 95.3 [94.3-96.4] | 95.7 [94.3-97.0] | 95.5 [94.5-96.4] | 97.1 [96.3-97.8] |
| Micro average | 2822 | 97.7 [97.4-98.1] | 96.1 [95.4-96.9] | 96.8 [96.2-97.5] | 96.5 [95.9-97.0] | 97.4 [97.0-97.8] |
| test set D (N=500) | | | | | | |
| Infiltrates | 105 | 94.6 [92.8-96.6] | 86.1 [79.4-92.4] | 88.6 [83.0-94.3] | 87.3 [82.8-91.8] | 92.4 [89.4-95.4] |
| Congestion | 176 | 97.8 [96.4-99.0] | 97.1 [94.5-99.4] | 96.6 [93.6-98.9] | 96.9 [94.9-98.5] | 97.5 [95.9-98.8] |
| Effusion | 241 | 99.4 [98.6-100] | 100 [100-100] | 98.8 [97.2-100] | 99.4 [98.6-100] | 99.4 [98.6-100] |
| Pneumothorax | 20 | 99.4 [98.6-100] | 100 [100-100] | 85.0 [66.7-100] | 91.9 [80.0-100] | 92.5 [83.3-100] |
| Regular CVC | 211 | 95.8 [94.0-97.4] | 93.6 [90.1-96.6] | 96.7 [94.0-99.0] | 95.1 [92.9-97.0] | 95.9 [94.2-97.5] |
| Misplaced CVC | 50 | 98.0 [96.6-99.0] | 87.0 [78.1-95.5] | 94.0 [86.7-100] | 90.4 [84.3-95.5] | 96.2 [92.5-99.2] |
| Macro average | 803 | 97.5 [96.9-98.1] | 94.0 [92.2-95.8] | 93.3 [89.7-96.1] | 93.5 [91.0-95.3] | 95.7 [93.8-97.1] |
| Micro average | 803 | 97.4 [96.8-98.1] | 95.1 [93.8-96.4] | 95.8 [94.2-97.2] | 95.4 [94.3-96.5] | 96.8 [95.9-97.6] |

**Table 3.** Accuracy, recall, precision, F1-scores and AUC (%) for each class on both test set C and D for T_mlm,gold_ trained with all available data. Support (SP) of all classes on second test set for comparison. Note that the 95% confidence intervals were obtained by an additional run of bootstrapping with 1000 resamples with a different random seed, which results in slight differences of F1-score intervals on test set C compared to Table 1.

**References**

1. Nakayama H, Kubo T, Kamura J, Taniguchi Y, Liang X (2018) doccano: Text Annotation Tool for Human. Available via https://github.com/doccano/doccano. Accessed 28 Jul 2022
2. Bird S, Klein E, Loper E (2009). Natural language processing with Python: analyzing text with the natural language toolkit. O'Reilly Media, Inc.
3. NLTK Snowball Stemmer. Available via https://www.nltk.org/_modules/nltk/stem/snowball .html Accessed 28 Jul 2022
4. Wu Y, Schuster M, Chen Z et al (2016) Google's neural machine translation system: Bridging the gap between human and machine translation. arXiv preprint arXiv:1609.08144
5. Wolf T, Debut L, Sanh V et al (2019) Huggingface's transformers: State-of-the-art natural language processing. arXiv preprint arXiv:1910.03771
6. Deepset (2021) German BERT. Available via https://huggingface.co/bert-base-german-cased. Accessed 28 Jul 2022
7. Shrestha M (2021) German Medical BERT. Available via https://huggingface.co/smanjil/German-MedBERT. Accessed 28 Jul 2022
